# Health Status of the Clinical Dental Students in the Jordanian Universities

**DOI:** 10.4021/jocmr2009.04.1233

**Published:** 2009-04-21

**Authors:** Darwish Badran, Ramzi Duaibis, Muna Al-Ali, Tamara Oweis, Walaa Amin

**Affiliations:** a Center for Educational Development, University of Jordan, Amman, Jordan,; b Faculty of Dentistry, University of Jordan, Amman, Jordan.

## Abstract

**Background:**

Dental students are subjected to many stresses that may affect their achievement. The purpose of this study is to assess the mental and physical health of dental students in two Jordanian Universities.

**Methods:**

A total of 265 dental students and 228 non-dental students from two Jordanian Universities participated in the study. They completed the survey questionnaire and their responses were used in calculating the 0-100 scores for the eight health concepts by linear transformations of scores. The ANOVA test was used to determine the significant differences among the student groups, and Tukey test was used for multiple comparisons among groups. All tests were carried out at 95% confidence level.

**Results:**

The results indicated that the dental students of the Jordan University of Science and Technology were of better health than their counterparts at the University of Jordan. The health scores attained by the dental students of the two universities were less than those of non-dental students of the same age.

**Conclusions:**

The physical and more significantly the mental health components of dental students should receive more attention, and further work is needed to detect the possible causes and find potent remedies for this problem.

**Keywords:**

Health survey; Clinical students; Physical functioning; Mental health; Social functioning

## Introduction

Practicing dentistry poses many occupational health hazards, including musculoskeletal problems, infections, and mental symptoms [1, 2, 3]. The relation between mental and physical health problems has been proven [4]. Stress can cause many physical complaints, such as neck pain, headaches, and backaches [5]. Some of these health problems can result from the work environment that requires certain postures and imposes certain mental and psychological challenges that have to be endured [1, 4, 5]

In some reported surveys, dentists were asked to assess their own health. Many regarded their health negatively and attributed that to their work [4, 6]. Missing work days was frequently regarded to work related illness [6]. Also, health behavior of dentists was work related [4, 7, 8]. Alcohol abuse was considered as a mean to ease the stress out [2, 4, 6]. Sedentary life style, was primarily considered due to having little or no time [4, 7]

Since dental students work in a similar environment to that of dentists, their health was studied too, but unfortunately with much less intensity [8, 9, 10, 11]. Dental students are continuously subject to the pressure of meeting the demands of learning, patients and supervisors expectations, and navigating through their own lives' demands.

A study on seven European dental schools revealed that almost one third of the students reported psychological distress [9]. Physical health of female students was found to be less positive than that of male students [9]. Pohlmann et al, on the other hand, found students' health close to normal, in all three German dental schools included in the study [8]. A third study [10] found that upper body musculoskeletal symptoms are more common in dental students of two schools than psychology students, and that these symptoms, especially lower back symptoms, were present even though students were not subject to the same work load as dentists.

The original version of SF-12 health survey was developed in 1994 as a short version of the SF 36 health survey [12, 13].The second version of SF12 Health Survey was developed recently, in order to increase precision through improving the structure of the survey. The improvement included the instructions, layout, comparability with translations and the adoption of five-level response choices [14].

In Jordan, there are two dental schools, one in the capital city, Amman, the other in the northern city of Irbid. The two dental schools have different collegiate environments and teaching styles. The students of both dental schools have more intense curricula than non-dental students of the same universities. It is expected, therefore, that these curricula of different intensities could have different implications on the health status of the corresponding students.

The objective of this study was to assess the health of dental students in Jordan universities, both physically and mentally using the SF12-v2 Health Survey, and to compare the results with those of non dental students of the same universities and with those reported for other universities.

## Materials and Methods

The general health of Jordanian dental students was assessed by employing the second version of SF12 health survey. This survey covered eight health scales (Physical Functioning, PF; Role Physical, RP; Bodily Pain, BP; General Health, GH; Vitality, VT; Social Functioning, SF; Role Emotional, RE; and Mental Health, MH). Each of these health scales was represented in the survey by one or two items. Physical Function items were concerned with the one's ability to do typical daily moderate activities, for example, climbing up the stairs. Role physical is concerned with the effect of one's health on doing regular daily activities. Bodily pain measures the extent to which pain affects one's daily life. A general evaluation of one's health is what the general health scale measures. Vitality measures how energetic the person feels. Social functioning is basically how much the person's health interferes with one's social life. Role Emotional is a measure of the extent to which one's emotions or mental status affects daily interactions or regular activities. Mental health measures the mental status of the person.

One hundred forty eight dental students (83 fourth year and 65 fifth year) from the University of Jordan (UJ) in the capital city Amman and 117 dental students (51 fourth year and 66 fifth year) from the Jordan University of Science and Technology (JUST) in the northern city of Irbid participated in the survey. The survey also included non-dental students from the same universities (100-non dental students from UJ and 128 students from JUST to provide baseline levels for each university.

The questionnaire was handed to the students during a lecture after the mid-term exams of the second semester (2007). Participation of the students was anonymous and voluntary. The responses of each student were re-coded and transformed to a 0-100 scale values for the eight health concepts as explained in the User's Manual for the SF12-v2Health Survey.

Normal values for the Jordanian population are not available, which made the use of Norm-based scoring and summary scales not feasible. Previously reported studies that used the Norm-based scoring system investigated samples of populations whose Norm-based values were known. Normbased scoring is usually achieved by linear transformations of scores to calculate a mean of 50 and a standard deviation of 10 for all eight health scales and both physical and mental health summary measures. This transformation has been and still applied to all SF12-v2 Health Survey scales to facilitate interpretation and comparisons among population groups [14].

One way analysis of variance (ANOVA) was used in determining whether any significant differences existed among the student groups. Multiple comparisons among the different groups were performed using Tukey test. The various data sets were rigorously treated statistically at the 95 percent level of confidence. Statistical analysis was carried out using the Statistical Package for Social Sciences (Version 15.0, SPSS Inc., Chicago, Illinois, USA).

## Results

**Figure 1:**
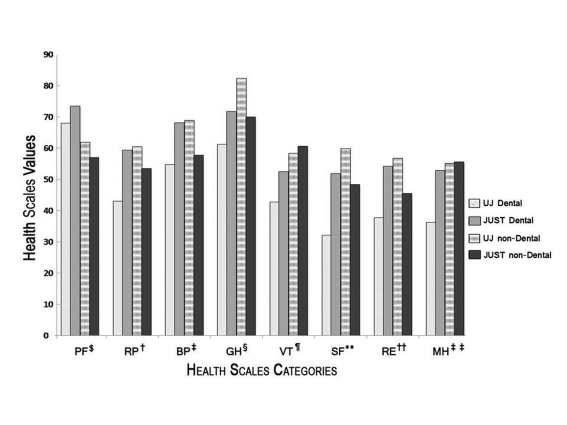
A Histogram comparing the scores of the eight health scales among the Dental student groups in the UJ and JUST, and between them and the scores of the non-dental student groups in the two universities. PF, Physical Functioning; RP, Role Physical; BP, Bodily Pain; GH, General Health; VT, Vitality; SF, Social Functioning; RE, Role Emotional; MH, Mental Health

Mean values for the eight health scales for each student group were presented in Table 1. ANOVA test revealed that significant differences existed among different groups (Table 2). Tukey test, however, showed no significant differences between the 4th and 5th year students of the same university in all of the eight health scales (Table 2, Fig.1). The students of the 4th and 5th year of each dental school were, therefore, clustered together and considered as one group for reasons of comparisons with the various other groups. Significant differences were found between dental student groups of the two universities in seven of the eight health scales (Table 2, Fig.1).

**Table 1:** Mean Score for all student groups for all health scales

Group	PF	RP	BP	GH	VT	SF	RE	MH
**UJ**								
4th Year	66.87	43.37	56.63	59.04	40.66	28.61	36.30	37.50
5th Year	69.62	42.69	52.69	64.23	45.77	36.92	39.81	34.81
4th-5th Year	68.07	43.07	54.90	61.32	42.91	32.26	37.84	36.32
Non-Dental	62.00	60.63	69.00	82.50	58.52	60.00	56.88	55.25
**JUST**								
4th Year	74.02	56.13	72.55	73.04	55.88	53.92	50.00	52.70
5th Year	73.11	61.93	64.77	70.83	50.00	50.38	57.58	53.22
4th-5th Year	73.50	59.40	68.16	71.79	52.56	51.92	54.27	52.99
Non-Dental	57.03	53.61	57.81	70.04	60.74	48.44	45.51	55.66

PF, Physical Functioning; RP, Role Physical; BP, Bodily Pain; GH, General Health; VT, Vitality; SF, Social Functioning; RE, Role Emotional; MH, Mental Health

**Table 2:** P values as determined by Tukey test between the student groups for all SF 12v2 subscales at 95% confidence interval

Student Group	PF	RP	BP	GH	VT	SF	RE	MH
UJ 4th year - UJ 5th year	0.995	1	0.947	0.662	0.833	0.508	0.953	0.971
JUST 4th year - JUST 5th year	1	0.801	0.616	0.993	0.819	0.986	0.553	1
UJ dental - JUST dental	0.485	0.000*	0.000*	0.000*	0.013*	0.000*	0.000*	0.000*
UJ Dental - UJ non-dental	0.425	0.000*	0.000*	0.000*	0.000*	0.000*	0.000*	0.000*
JUST dental - JUST non-dental	0.000*	0.252	0.013*	0.913	0.061	0.782	0.026*	0.75

PF, Physical Functioning; RP, Role Physical; BP, Bodily Pain; GH, General Health; VT, Vitality; SF, Social Functioning; RE, Role Emotional; MH, Mental Health

## Discussion

The dental faculties in the two Jordanian Universities (UJ and JUST) offer a five-year program leading to a BDS degree. English is the teaching language, and both faculties followed a classic academic curriculum which dedicated the first three years to basic sciences and the last two years to clinical training.

The results of the present investigation indicated that the 4th and 5th year students of each university had approximately equal scores for all health scales with no significant differences between them. Whereas, the health scores of the students of Dentistry were significantly different from those of their non-dental counterparts in the same university.

These findings may reflect an overall condition which applied to all clinical dental students of the same university and indicating that these students were exposed to similar external factors related to the teaching and clinical training they received and probably to a comparable living atmosphere.

The scores of the clinical students at JUST were consistently higher than those of their counterparts in the UJ. This may be attributed to the fact that JUST is located in the northern city of "Irbid", a smaller city than the capital Amman in both area and population. It is known that life in small cities and towns is less stressful or demanding than in larger cities. Although this cannot be thought of as the single cause of these findings, nevertheless, it probably contributed to the "better health" of the JUST students.

There are several other differences that existed between the two dental schools regarding the clinical training program that might have had a positive influence on the health status and the well being of the JUST dental students. The dental faculties of both universities adopted a clinical training system which obligated a student to successfully finish a minimum amount of clinical requirements in order to pass any clinical course. Nevertheless, the minimum clinical requirements of the UJ dental faculty were much more in terms of quantity than those of JUST Dental students. This was expected to have a stressful effect on the mental as well as the physical health status of the UJ students more than what was encountered by their counterparts in JUST. This effect could have, probably, been exacerbated by the fact that the dental faculty of the UJ did not possess a patient-records filing system which meant that the students were required to find their own patients throughout their clinical training. The dental faculty at JUST, on the other hand, organized their patients' records and distributed them equally to the students based on their needs.

Students at JUST had less frequent clinical sessions per week compared to the UJ students. Going more frequently to the clinic demanded more physical and mental efforts, and the short sessions posed an additional stress on students who had to race against time to get their clinical task correctly executed and endorsed by their supervisors within the allocated time frame.

The staff/student ratio was more favourable in JUST than in the UJ. This had reduced the stress and the efforts associated with clinical training and improved the learning experience of the students by increasing the availability of the teaching staff during the clinical sessions. In addition, JUST provided an Allied Dental Sciences program whose students assisted the dental students during their clinical sessions, putting four-handed dentistry into practice. Dental students of the UJ on the other hand, worked alone during their clinical sessions since UJ did not provide a training program for dental hygienists and assistants.

Dental laboratory work was the responsibility of the dental students of UJ who had to send their laboratory work to laboratories outside the university or did the work themselves, while JUST allocated a dental technician to a number of clinical students to help them accomplish their laboratory procedures.

Moreover, JUST dental students worked in a dental center dedicated for clinical training and lectures all at one place. Whereas, UJ dental students attended their lectures at the Dental faculty while the clinical sessions were held at the University's hospital.

Non-dental students of the UJ demonstrated significantly higher health scores than their dental counterparts except for PF (physical function), where no significant difference existed. This could be attributed to the fact that the dental students are held at pressure all along their study years. Dental students had more credit hours required for graduation. These credit hours were distributed onto each semester as packages which resulted in having all semesters with a full hour load. On the other hand, non-dental students had less credit hours required for their graduation and these were distributed along the semesters in a more flexible fashion compared to the curricula of the health science studies. Adding to the aforementioned differences, the dental students were responsible for clinical requirements and patient handling with the resultant emotional exhaustion and its consequences [15].

JUST students had closer scores than did their UJ counterparts. Some significant differences were found between JUST student groups; in PF, BP, and RE. Dental students scored higher than their non dental counterparts in these scales. This may be ascribed to the fact that the dental students attended their lectures and clinics in one place; the health centre. Non-dental students, on the other hand, attended their lectures in various faculties at the university campus and had to move relatively long distances in order to attend lectures.

A German study evaluated and compared the health of dental students in German universities with that of the general population found no significant differences between the dental students and German norms except for the mental health summary measure in a single university [8]. In our context, similar comparison was not possible because norms for Jordanian population do not exist because the SF12-v2 Health Survey has not been previously applied to the Jordanian population.

Further studies aimed at establishing norms for the Jordanian population are imperative. Regardless of the findings of such studies, the scores found in the present study were alarming and require serious attention and care.

Taking into consideration the results of this study, it seems that the stressful and demanding atmosphere created in clinical training has significant detrimental effects on the health and well-being of dental students. The deterioration noted in the physical and more significant in the mental health components of dental students should not be overlooked, but continuously monitored by the dental faculties who are supposed to detect the possible causes and find potent remedies for this serious problem.

In conclusion, dental students of the JUST are of better physical and mental health compared to their counterparts in the UJ; dental students in UJ scored lower health values, in general, than those of 18-24 year old non-dental students and consequently, have a poorer general health; the dental faculties at the both Jordanian universities should review and update their teaching curricula to minimize the physical and mental stresses encountered by the students; longitudinal studies applying the SF12-v2 health survey should be carried out in both universities to monitor the health of dental students, to find the possible causes of their health deterioration and consequently, find the cures; the SF12-v2 Health Survey should be applied on dental students in other countries to determine whether a generalized health deterioration problem exists among dental students or not. The SF12-v2 Health Survey should also be applied on the Jordanian population in a large-scale study in order to evaluate the general health of Jordanians, to establish national norms and compare them with other populations.
